# Different patterns of MRI structural alterations in schizophrenia spectrum disorders with delusions of influence vs persecution

**DOI:** 10.1192/j.eurpsy.2024.1287

**Published:** 2024-08-27

**Authors:** A. S. Tomyshev, A. Dudina, D. Romanov, E. Ilina, M. Magomedagaev, G. Kostyuk, A. Andriushchenko, A. Smulevich, I. Lebedeva

**Affiliations:** ^1^Laboratory of Neuroimaging and Multimodal Analysis, Mental Health Research Center; ^2^Department of Psychiatry and Psychosomatics, I.M. Sechenov First Moscow State Medical University; ^3^Department of borderline mental pathology and psychosomatic disorders, Mental Health Research Center; ^4^Mental-health Clinic No.1 named after N.A. Alexeev, Moscow, Russian Federation

## Abstract

**Introduction:**

There is strong evidence that delusions are associated with cortical and subcortical structural alterations. However, whether these abnormalities differ in different types of delusions within schizophrenia spectrum disorders remains unclear.

**Objectives:**

We aimed at exploring structural neural patterns underlying influence/persecutory delusions across diagnostic categories within the schizophrenia-spectrum disorders.

**Methods:**

Twenty six right-handed patients with delusional disorder (n=7) and schizophrenia (n=19), presenting with delusions were divided in two clinical subgroups. The first one was presented with persecutory delusions (n=12, 20.2-55.4 years, mean age 36.0±11.6, 2 females) and the second with delusions of influence (n=14, 21.2-47.6 years, mean age 36.7±8.4, 2 females). The control group consist of 26 matched healthy controls (20.2-53.6 years, mean age 36.3±9.9, 4 females). All participants underwent structural MRI at 3T scanner. MRI images were processed via FreeSurfer 6.0 to quantify cortical thickness (CTh) and volumes for subcortical and brainstem (midbrain, pons, superior cerebellar peduncle and medulla) structures.

**Results:**

Compared to healthy controls, patients with delusions of influence showed decreased gray matter thickness in 17 cortical regions (Figure, A), and decreased volumes of thalamus bilaterally (left: F(1,35)=9.0, p=0.005; Cohen’s *d*=−0.8; right: F(1,35)=8.0, p=0.008; Cohen’s *d*=−0.8), left hippocampus (F(1,35)=8.8, p=0.005; Cohen’s *d*=−0.8), midbrain (F(1,35)=11.0, p=0.002; Cohen’s *d*=−0.9) and pons (F(1,35)=8.2, p=0.007; Cohen’s *d*=−0.8). Conversely, patients with delusions of persecution showed decreased thickness only in 4 cortical regions of the right hemisphere (figure, B) and no alterations in subcortical volumes compared to healthy controls.

No differences in cortical or subcortical measures between two clinical subgroups survived corrections for multiple comparisons. No correlations between structural alterations and total PANSS or BABS scores were found in either clinical groups.

Figure. Clusters of decreased cortical thickness according to atlas of Desikan et al. (2006) in delusions of influence (A) and delusions of persecution (B) subgroups compared to healthy controls.

**Image:**

**Figure:**
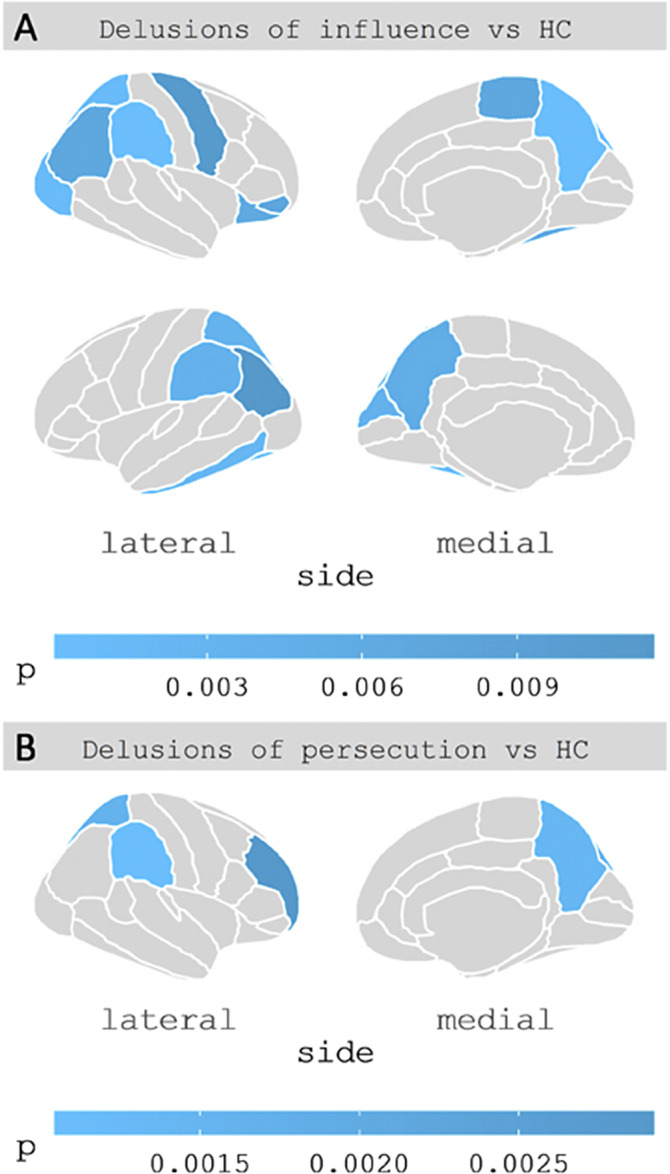

**Conclusions:**

The findings suggest that patients with delusions of influence have more pronounced cortical, subcortical and brainstem structural deficit as compared to patients with delusions of persecution. However, the limited sample size and the lack of correlations with clinical scores do not allow to conclude definitely whether the revealed structural abnormalities underlies delusions of influence, which should be elucidated via further research.

*This study was supported by RFBR grant 21-515-12007*

**Disclosure of Interest:**

None Declared

